# A comparison of ultrasound and clinical examination in the detection of flexor tenosynovitis in early arthritis

**DOI:** 10.1186/1471-2474-12-91

**Published:** 2011-05-08

**Authors:** Ihsane Hmamouchi, Rachid Bahiri, Najlaa Srifi, Souad Aktaou, Redouane Abouqal, Najia Hajjaj-Hassouni

**Affiliations:** 1 Laboratory of Information and Research on Bone Diseases (LIRPOS). Department of Rheumatology, University Mohammed V Souissi, Faculty of Medicine and Pharmacy, El Ayachi hospital, University Hospital of Rabat-Sale, Morocco; 2 Laboratory of Biostatistical, Clinical and Epidemiological Research (LBRCE). University Mohammed V Souissi, Faculty of Medicine and Pharmacy, Rabat, Morocco

## Abstract

**Background:**

Tenosynovitis is widely accepted to be common in rheumatoid arthritis (RA) and postulated to be the first manifestation of RA, but its true prevalence in early disease and in particular the hand has not been firmly established. The aims of this study were first to investigate the frequency and distribution of finger flexor tenosynovitis using ultrasound in early arthritis, second to compare clinical examination with ultrasound (US) using the latter as the gold standard.

**Methods:**

33 consecutive patients who had who were initially diagnosed with polyarthritis and suspected of polyarthritis and clinical suspicion of inflammatory arthritis of the hands and wrists were assessed during consecutive, routine presentations to the rheumatology outpatient clinic. We scanned a total of 165 finger tendons and subsequent comparisons were made using clinical examination.

**Results:**

Flexor tenosynovitis was found in 17 patients (51.5%) on ultrasound compared with 16 (48.4%) of all patients on clinical examination. Most commonly damaged joint involved on US was the second finger followed by the third, fifth, and fourth. Both modalities demonstrated more pathology on the second and third metacarpophalangeal (MCP) compared with the fourth and fifth MCP. A joint-by-joint comparison of US and clinical examination demonstrated that although the sensitivity, specificities and positive predictive values of clinical examination were relatively high, negative predictive value of clinical examination was low (0.23).

**Conclusions:**

Our study suggest that clinical examination can be a valuable tool for detecting flexor disease in view of its high specificity and positive predictive values, but a negative clinical examination does not exclude inflammation and an US should be considered. Further work is recommended to standardize definitions and image acquisition for peritendinous inflammation for ultrasound.

## Background

Tenosynovitis is widely accepted to be common in RA and postulated to be the first manifestation of RA [1], but its true prevalence in early disease and in particular the hand has not been firmly established.

Several studies have previously highlighted the ability of US for detecting tendon disease in the RA hand [2-6], and some have described US as the gold standard imaging method for assessing tendon involvement in rheumatic diseases [7]. It's defined by abnormal hypoechoic or anechoic material with or without fluid inside the tendon sheath and with possible signs of Doppler signals in two perpendicular planes [8]. It may be caused by invasion of pannus into the tendon or by pannus due to compression, both causing oedema, ischemia and necrosis [9,10]. A serious complication of persistent tenosynovitis is complete rupture of the tendon with loss of finger function [10].

Early diagnosis of joint inflammation and early institution of immunosuppressive drug treatment -- targeted at reducing synovial inflammation -- is now being advocated in order to prevent subsequent joint damage and disability [11]. Despite the importance of tendon disease for hand function, it is essential therefore that tendon disease can be accurately assessed, particularly in early disease.

The present study has two objectives: First, To investigate the frequency and distribution of finger flexor tenosynovitis using US in early unspecified arthritis or suspected RA, second to compare gray-scale clinical examination with US using the latter as the gold standard.

## Methods

### 1. Patients

The local research ethics committee approved the study protocol and all patients gave informed written consent prior to their inclusion in the study. 33 consecutive patients who has originally presented with polyarthritis and clinical suspicion of inflammatory arthritis of the hands and wrists (symptoms < 24 months) were assessed during consecutive, routine presentations to the rheumatology outpatient clinic. In all patients, the screening was performed by HI and SN, the clinical examination by AS and US by BR. For the purpose of the study, US was performed by BR without knowledge of the clinical score assigned by AS or the tendon assessment. As a rule, the clinical examinations and US were performed on the same day.

### 2. Clinical examinations

Clinical examinations were performed by a senior rheumatologist trained in the detection of musculoskeletal disorders (AS), who disregarded ultrasonography findings. Typical symptoms of flexor digitorum tenosynovitis were defined according to the Birmingham consensus criteria [12]. Thus, in each wrist and finger, symptoms were assessed in terms of volar pain involving the hand, wrist or forearm during active movement of the tendon against resistance, including pinching and grasping. A binary scoring system (0-1) was used to assess each tendon as normal (0) or abnormal (1) for tenderness, crepitus and swelling. We studied 5 sites per hand: the wrist, and the second, third, fourth, and fifth finger for signs of tenosynovitis. Repetitive use of an extremity often precipitates tenosynovitis. We wanted to eliminate the causes of degenerative tenosynovitis that could occur at the dominant hand. Only lesions in the non-dominant hand were taken into account therefore 165 flexor tendons were included in the study.

### 3. Ultrasound evaluation [8]

For the screening of arthritic joint processes, the following procedures were used:

1. Longitudinal and transverse scan of the wrist (dorsal, ulnar, palmar aspect) for signs of tenosynovitis.

2. Longitudinal and transverse scan of the MCP joints and the PIP joints II-V (dorsal, palmar aspect) for signs of synovitis, tenosynovitis/tendinitis.

All gray-scale scans were performed using a HITACHI machine with a 7.5 - 13 MHz linear array transducer. Gel was used to provide an acoustic interface. One sonographer (RB) sequentially and independently performed scans on each patient. Each joint was scanned across both volar and dorsal aspects in longitudinal and transverse planes to provide maximum coverage of the joint and avoid artefacts.

**Figure 1:**
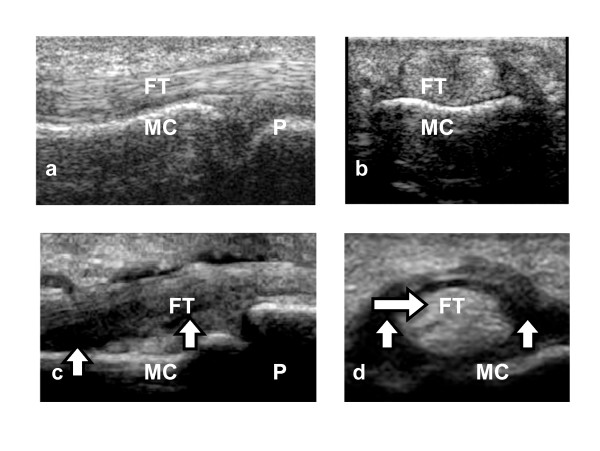
**Ultrasound appearance of normal flexor tendon sheath and tenosynovitis**. a, Normal appearance, longitudinal view. b, Normal appearance, transverse view. c, Flexor tenosynovitis, longitudinal view. d, Flexor tenosynovitis, transverse view. Arrows in c and d indicate tendon sheath thickening. MC: metacarpal; P: phalanx; FT: flexor tendon

The synovial sheath of the flexor tendon, which was identified as a slightly hypoechoic area, was clearly detectable at the edge of the tendon's profile on the transverse scans. The presence of a well-defined area of increased echogenicity within the tendon sheath was considered to indicate synovial thickening. The presence (1) or absence (0) of flexor tenosynovitis (Figure 1) was documented.

### 4. Intra-reader reliability

Random ultrasounds from 10 patients were re-performed by a single experienced reader (BR).

### 5. Statistical analysis

As a first step, we investigate the prevalence of flexor tenosynovitis in early arthritis. Then, we tried to conduct a multivariate analysis to detect the factors associated with the existence of tenosynovitis like tender joint count, swollen joint count, presence of erosions. However, this approach did not result in any relevant association. Finally, agreement statistics were used to calculate the sensitivity, specificity, positive predictive value, and negative predictive value for clinical examination using US as the gold standard. Kappa values were calculated for intra-reader reliability. All statistical analyses were carried out using SPSS 13.0 (SPSS, Chicago, IL).

## Results

### 1. Demographics

Thirty-three patients were included in the study. The patient characteristics are represented in Table 1. The mean age was 43 years and the mean duration of disease was 52 weeks.

**Table 1:** Clinical and demographical data of patients with early arthritis

Variable	Values
Number of patients	33
	Median (IQR)
Age, years	43 (27-53)
DAS 28	5.8 (4.6-6.8)
HAQ	0.8 (0.5-1.2)
Duration of the disease, weeks	52 (16-65)
VAS of disease activity	50 (30-80)
Early morning stiffness (min)	45 (25-120)
Joint count for swelling	5 (4-10)
Joint count for tenderness	9 (6-14)
ESR (mm)	35 (28-66)
CRP (mg/dl)	20 (12.5-35)

	N (%)
Femal	22 (66.7)
Education level	
No formal education	5 (15.2)
Primary school	10 (30.3)
Secondary school	11 (33.3)
High school	8 (21.2)
Rhematoid factor	15 (45.5)
Diagnostic	
Rheumatoid arthritis	20 (60.6)
Undifferentieted oligoarthritis	13 (39.4)

IQR: interquartiles ranges; NSAIDs: non steroidal antiinflammatory drugs; ESR: erythrocyte sedimentation rate; CRP: C-reactive protein

Drugs at entry included non-steroidal anti-inflammatory drugs (33.3% of patients), corticosteroids (97%), methotrexate (30.2%), sulfasalazine (6%). One patient received no drugs.

We scanned a total of 165 finger tendons and subsequent comparisons were made using clinical examination.

### 2. Clinical and Ultrasound evaluation

**Figure 2:**
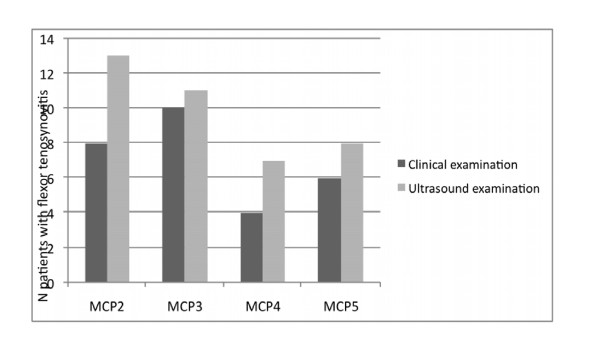
**Distribution of flexor tenosynovitis across the metacarpophalangeal (MCP) joint using ultrasound (US) and clinical examination**.

Flexor tenosynovitis was found in 17 patients (51.5%) on US compared with 16 (48.4%) of 33 patients on clinical examination. The distribution of joints involved using each modality is represented in Figure 2. Tenosynovitis is more frequently found the second finger and the third, fifth, and fourth. Both modalities demonstrated more pathology on the second and third MCP compared with the fourth and fifth MCP. The agreement statistics between clinical examination and US (used as the gold standard) are presented in Table 2 and 3.

**Table 2:** Comparison of Clinical examination and US (gold standard) for the detection of flexor tenosynovitis

Clinical examination versus US (gold standard)						
	**Sensitivity**	**Specificity**	**PPV**	**NPV**	**LRP**	**LRN**

Tenderness	0.87	0.47	0.61	0.20	1.65	0.26
Crepitus	0.56	0.76	0.69	0.35	2.39	0.57
Swelling	0.25	1	1	0.41	infinity	0.75
At least one symptom	0.93	0.41	0.60	0.12	1.59	0.15
At least two symptons	0.68	0.82	0.78	0.26	3.89	0.37
All three findings	0.06	1	1	0.46	infinity	0.93
Specialist diagnostic	0.75	0.76	0.75	0.23	3.18	0.32

PPV: positive predictive value, NPV: negative predictive value, LRP: likehood ratio positive, LRN: likehood ratio negative

**Table 3:** US and clinical examination in the detection of tenosynovitis of finger flexor tendons in 33 patients with early rheumatoid arthritis

Tenosynovitis		Ultrasound examination	
Clinical examination		Positive	Negative
Tenderness	Positive	14	9
	Negative	2	8

Crepitus	Positive	9	4
	Negative	7	13

Swelling	Positive	4	0
	Negative	12	17

At least one symptom	Positive	15	10
	Negative	1	7

At least two sympton	Positive	11	3
	Negative	5	14

All three findings	Positive	1	0
	Negative	15	17

Specialist diagnostic	Positive	12	4
	Negative	4	13

### 3. Intra-reader reliability

The kappa values for the detection of tendon disease in 40 joints from 10 patients were 0.85 and 0.8 for US and clinical examination respectively.

## Discussion

Flexor tenosynovitis was found in 17 patients (51.5%) on US compared with 16 (48.4%) of 33 patients on clinical examination. The intra-reader reliability of reading both the US and clinical examination was good (kappa = 0.8). A joint-by-joint comparison of US and clinical examination demonstrated that although the sensitivity, specificities and positive predictive values of clinical examination were relatively high, negative predictive value of clinical examination was low (0.23).

There are limited published data on patients with early disease and very few using imaging in the hand. The reported prevalence of flexor tenosynovitis in RA is mainly based on studies involving clinical examination of patients with longstanding RA and varies from 5% to 55% [13]. In a study of 60 patients with inflammatory arthritis [14], US was found to detect tendon sheath widening in 21% of flexor tendons and 5% of extensor tendons. Another study demonstrated a high frequency of flexor tenosynovitis seen on MRI and US (64% joints, versus 28.5% joints) [15].

The present study demonstrated a high frequency of flexor tenosynovitis seen on US and clinical examination in early arthritis. Larger studies are required to provide final conclusion.

The differences between US and clinical examination may reflect differences in level examinations. In fact, the US examinations performed at the level of the MCP joint, whereas in clinical practice the transducer is often moved up and down the length of the tendon, with the tendon flexed and extended to enable further clues that might suggest inflammation.

Ultrasonography had higher sensitivity for detecting signs of inflammation in the examined finger joints than did clinical examination [8]. Predictably enough, in our data the sensitivity, specificities and positive predictive values of clinical examination were relatively high, but negative predictive value of clinical examination was low (0.23).

The distribution of joint pathology is poorly described in the literature, particularly in early disease. The preponderance of flexor tenosynovitis on the third MCP joint is consistent with the clinical finding by, Gray and Gottlieb that this was the most frequent site of tenosynovitis, although their patients had a longer duration of disease (mean 5 years) [13]. The predisposition toward the second and third joints probably relates to biomechanical factors such as relative increased range of movement of these joints [16] as has been suggested for the reason of a higher prevalence of bone erosion and synovitis [17] and has been showed in studies comparing US and MRI.

Our study has strengths and some limitations. First, because arthritis may be characterised by phases of flares and respite, the results we observed might be slightly overestimated compared with the whole population of patients. Then, we did not include power Doppler but it was thought unlikely that it would have increased the sensitivity of US because Doppler signals rarely occur in tendon sheaths that are normal on gray scale [wed]. However, the growing number of reports comparing Doppler ultrasonography with MRI [18,19] and histology of joints [20,21], and describing the advantages of supporting ultrasonography with Doppler evaluation suggests that it will soon become a routine aspect of the joint assessment. Nonetheless, many methodological and technical aspects of the use of Doppler ultrasonography remain to be clarified [22].

## Conclusions

Tendon sheath inflammation was shown to be common using each modality, although US was more sensitive. The data suggests that clinical examination can be a valuable tool for detecting flexor disease in view of its high specificity and positive predictive values, but a negative clinical examination does not exclude inflammation and an US should be considered. Further work is recommended to standardize definitions and image acquisition for peritendinous inflammation for US.

## Competing interests

The authors declare that they have no competing interests.

## Authors' contributions

RB and NHH conceived the study and supervised its design, execution, and analysis and participated in the drafting and critical review of the manuscript. IH and RA did data management and statistical analyses. All authors participated in critical revision of the manuscript. IH wrote the paper with input from all investigators. All authors read and approved the final manuscript.

## Pre-publication history

The pre-publication history for this paper can be accessed here:

http://www.biomedcentral.com/1471-2474/12/91/prepub
